# Preclinical Evaluation of Atorvastatin-Loaded PEGylated Liposomes in a Mouse Model of Traumatic Brain Injury

**DOI:** 10.3390/ijms262412176

**Published:** 2025-12-18

**Authors:** Eun-Sol Hwang, Ja-Hae Kim, Ji-Hye Kim, Raveena Nagareddy, Yong-Yeon Jeong, Kang-Ho Choi

**Affiliations:** 1 Department of Biomedical Sciences, Chonnam National University Medical School and Hospital, Gwangju 61469, Republic of Korea; dmsthf2386@naver.com (E.-S.H.);; 2 Department of Nuclear Medicine, Chonnam National University Medical School and Hospital, Gwangju 61469, Republic of Korea; jhbt0607@hanmail.net; 3 Department of Neurology, Chonnam National University Medical School and Hospital, Gwangju 61469, Republic of Korea; 96248104@hanmail.net; 4 Department of Radiology, Chonnam National University Medical School and Hwasun Hospital, Hwasun 58128, Republic of Korea

**Keywords:** blood–brain barrier, nanoparticle, neuroinflammation, statin, traumatic brain injury

## Abstract

Evidence on the therapeutic use of nanoparticles for traumatic brain injury (TBI) remains limited. This study aimed to evaluate the neuroprotective potential of atorvastatin-loaded polyethylene glycol (PEG)-conjugated liposomes (LipoStatin) in a mouse model of repetitive TBI. TBI was induced using five controlled head impacts with a 120 g weight at 12-h intervals. Mice were randomly assigned to Sham, Control (saline-treated), Statin (free atorvastatin), Liposome (empty PEGylated liposomes without atorvastatin), and LipoStatin (atorvastatin-loaded PEGylated liposome) groups. LipoStatin (10 mg/kg/day) was intravenously administered for 5 days post-injury. Neurological function was evaluated using the neurological severity score (NSS), while blood–brain barrier (BBB) integrity and neuroinflammation were assessed on day 5, and cellular apoptosis on day 12. LipoStatin-treated mice exhibited the lowest NSSs. IVIS^®^ imaging indicated significantly attenuated BBB disruption (*p* < 0.001), and Western blot analysis revealed restored caveolin-1 protein levels (*p* < 0.01), which are associated with BBB integrity. TNF-α levels were reduced considerably in the LipoStatin group compared to both the Control (*p* < 0.001) and Statin (*p* < 0.05) groups. Immunofluorescence showed reduced co-localization of caspase-3 with PDGFR-β and GFAP, indicating decreased pericyte and astrocyte apoptosis. These findings suggest that LipoStatin may confer neuroprotection in TBI by stabilizing BBB integrity, reducing inflammation, and mitigating cell death, supporting its potential as an improved nanocarrier-based therapeutic approach.

## 1. Introduction

Traumatic brain injury (TBI) is one of the leading causes of mortality and disability; it affects a substantial portion of the population and has the highest incidence of all common neurological disorders [1,2]. It can lead to various short- or long-term health problems. TBI, even a mild injury, causes substantial neurological disabilities and mental distress temporarily or permanently. Notably, multiple concussions are less benign and can induce cumulative neurobehavioral impairments and neuropathological changes [2,3,4]. Repeated TBI is particularly frequent in athletes and military service members [5]. However, current management strategies for TBI remain primarily supportive, as no pharmacological interventions have improved short- or long-term functional outcomes [2,6].

Statins, which are 3-hydroxy-3-methyl-glutaryl-coenzyme A (HMG-CoA) reductase inhibitors that function as cholesterol-lowering drugs, have shown beneficial neuroprotective effects after various forms of brain injury, including ischemic stroke [7,8,9], TBI [10,11], and intracerebral hemorrhage [12,13,14]. They can suppress glial activation and reduce the secretion of inflammatory and oxidative stress mediators [15]. Their modulatory effects on neuroinflammatory responses may reduce secondary neuronal injury, blood–brain barrier (BBB) breakdown, and the development of cerebral edema [16,17]. Among statins, atorvastatin is considered more effective than hydrophilic agents against brain injury because of its ability to traverse the BBB [16,18]. Despite the therapeutic potential of atorvastatin, its efficacy in TBI may be limited by pharmacokinetic challenges and poor bioavailability at the lesion site, highlighting the need for improved delivery strategies.

Injury-mediated drug delivery approaches using nanoparticles can enhance the neuroprotective effects of statins by increasing drug exposure in regions of blood–brain barrier (BBB) disruption while reducing systemic adverse effects [19]. Recent studies have shown that nanoparticles can access injured brain tissue during transient increases in BBB permeability following TBI, allowing preferential accumulation at sites of neurovascular damage [20,21,22,23,24]. PEGylated liposomes are particularly advantageous due to their biocompatibility, biodegradability, and prolonged systemic circulation, which support passive accumulation within areas of injury-induced BBB breakdown [25,26,27,28]. Previous work evaluating LipoStatin in ischemic stroke models demonstrated superior therapeutic efficacy compared with conventional atorvastatin administration, suggesting potential benefits for neurovascular protection [16,29].

However, most previous studies on statin-loaded nanoformulations have focused on single-injury or ischemic models, without addressing the cumulative pathophysiological consequences of repetitive TBI. Moreover, the effects of nanoformulated statins on BBB-associated cellular mechanisms—particularly pericyte and astrocyte apoptosis—remain largely unexplored. To address these gaps, we evaluated the neuroprotective efficacy of PEGylated liposomal atorvastatin (LipoStatin) in a repetitive TBI mouse model, focusing on its ability to enhance delivery to injured brain regions, restore BBB integrity, and mitigate neuroinflammation and apoptosis in pericytes and astrocytes.

## 2. Results

### 2.1. Physicochemical Characterization of LipoStatin Formulation

**Figure 1:**
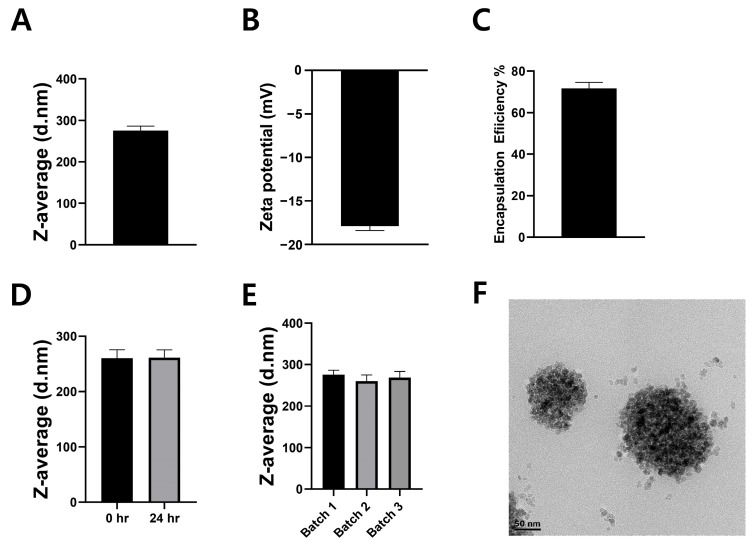
Physicochemical characterization of the LipoStatin formulation. (**A**) Hydrodynamic particle size (Z-average, d.nm) measured by dynamic light scattering. (**B**) Zeta potential (mV) of the LipoStatin formulation. (**C**) Encapsulation efficiency of atorvastatin within LipoStatin (%). (**D**) Stability evaluation of particle size after 24 h of storage at 4 °C. (**E**) Batch-to-batch reproducibility of particle size across three independent preparations (Batch 1–3). (**F**) TEM of liposomes loaded with synomag as a negative stain.

Physicochemical characterization was performed to evaluate the properties of the final LipoStatin formulation. The hydrodynamic size was 271 ± 15.44 nm with a polydispersity index (PDI) of 0.4 ± 0.03, and the zeta potential was –17.90 ± 0.87 mV, as determined by dynamic light scattering (Malvern Instruments, Malvern, UK). The atorvastatin encapsulation efficiency was approximately 71%, as determined by high-performance liquid chromatography (HPLC) [16]. Based on this encapsulation efficiency, nanoparticles were produced such that the amount of active atorvastatin contained in the formulation corresponded to 5 mg/kg. Stability assessment showed minimal changes in particle size after 24 h of storage at 4 °C, and batch-to-batch reproducibility was confirmed across three independent preparations (Figure 1A–E). Transmission electron microscopy revealed spherical, well-dispersed nanoparticles consistent with typical liposomal morphology (Figure 1F).

### 2.2. LC–MS Quantification of Atorvastatin Accumulation in Brain Tissue

**Figure 2:**
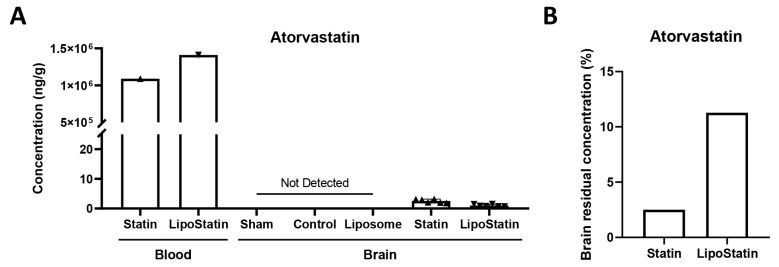
LC–MS quantification of atorvastatin exposure and brain retention. (**A**) Atorvastatin levels were measured in the injected drug solutions and in brain homogenates from each experimental group. Atorvastatin was detectable in the brain only in the Statin and LipoStatin groups. (**B**) Estimated percentage of the measured 1-h post-injection concentration of atorvastatin remaining in brain tissue 24 h later, reflecting the retention kinetics of free statin and LipoStatin.

To evaluate the extent of brain penetration following intravenous administration, LC–MS analyses were performed on both the administered drug solutions and brain homogenates collected from all experimental groups (Figure 2A). Analyses of the solutions in the blood confirmed high atorvastatin concentrations in both the Statin (~1.09 × 10^6^ ng/mL) and LipoStatin (~1.41 × 10^6^ ng/mL) formulations. In brain tissue, atorvastatin was not detected in the Sham, Control, or Liposome groups, indicating the absence of nonspecific background signal. In contrast, low nanogram concentrations were quantifiable in both the Statin and LipoStatin groups after 5 days of treatment, despite measurement 1 h after injection. Although the absolute concentrations remained low relative to the administered amount, these findings confirm successful passage of atorvastatin across the blood–brain barrier following systemic administration in the Statin and LipoStatin groups.

To compare retention dynamics in brain tissue, the residual concentration (%) was calculated as the estimated percentage of the measured 1-h post-injection concentration that remained 24 h later, just before the next dose. Based on this calculation, the Statin group retained approximately 2.5% of the initial detected level. In contrast, the LipoStatin group retained approximately 11.3% (Figure 2B), indicating the sustained-release and delayed distribution characteristics of the liposomal formulation.

### 2.3. Evaluating the Impact of Empty Liposomes on TBI Outcomes

To determine whether the liposomal vehicle itself influences biological responses following traumatic brain injury, we compared the Control and Liposome groups across multiple outcome measures, including BBB permeability, inflammatory gene expression, tight junction protein levels, and cell stress or apoptosis in pericytes and astrocytes.

**Figure 3:**
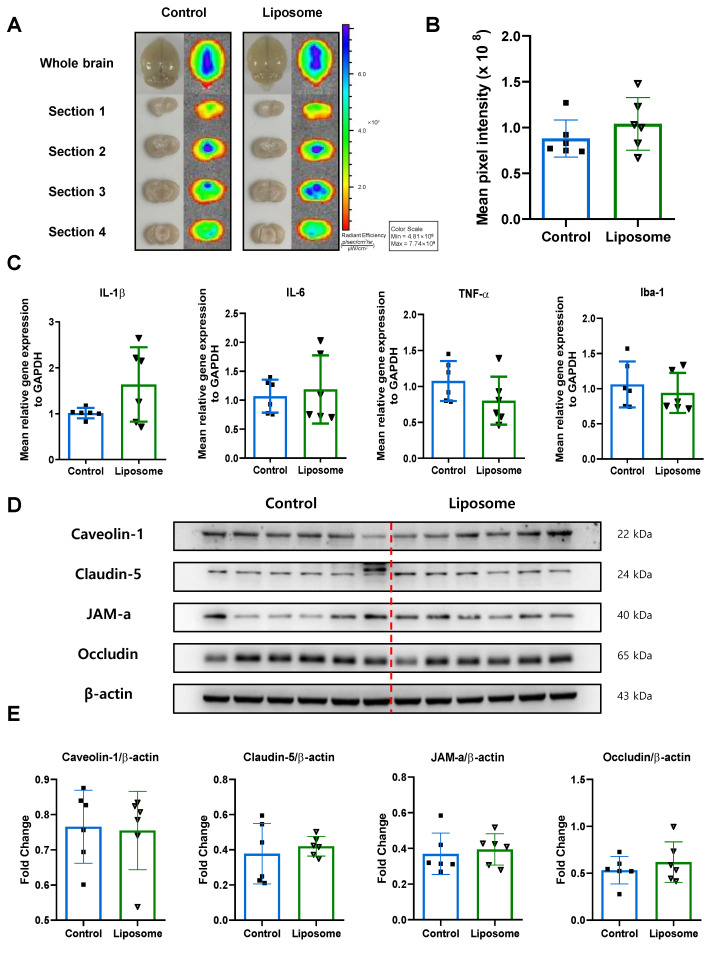
Evaluation of the impact of empty liposomes on BBB permeability, inflammation, and tight junction integrity following TBI. (**A**) Representative IVIS^®^ images of whole brains and four coronal sections showing Evans blue fluorescence in the Control and Liposome groups. (**B**) Quantification of mean pixel intensity indicates no significant difference in BBB leakage between groups. (**C**) qRT-PCR analyses of inflammatory markers (IL-1β, IL-6, TNF-α, Iba-1), showing no significant group differences. (**D**) Western blot analyses of tight junction–related proteins (Caveolin-1, Claudin-5, JAM-A, Occludin). (**E**) Quantification of tight junction protein levels normalized to β-actin.

IVIS^®^ imaging revealed that fluorescence intensity in the whole brain and across four coronal sections was comparable between the two groups, indicating no appreciable difference in BBB leakage (Figure 3A). Quantitative analyses further confirmed that the mean pixel intensity did not differ significantly, suggesting that empty liposomes do not affect BBB integrity (Figure 3B).

In the qRT-PCR analyses, the expression levels of key inflammatory markers (IL-1β, IL-6, TNF-α, and Iba-1) did not differ significantly between the Control and Liposome groups. Although minor fluctuations were observed in individual markers, no consistent trend was observed to support meaningful group-to-group variation. These findings indicate that empty liposomes do not exert measurable effects on inflammatory gene expression (Figure 3C).

Western blot analyses also demonstrated that the levels of representative tight junction proteins—Caveolin-1, Claudin-5, JAM-A, and Occludin—were similar between the two groups (Figure 3D). Densitometric quantification, normalized to β-actin, revealed no significant differences, suggesting that liposome administration does not alter the expression of structural proteins in the blood–brain barrier (Figure 3E).

**Figure 4:**
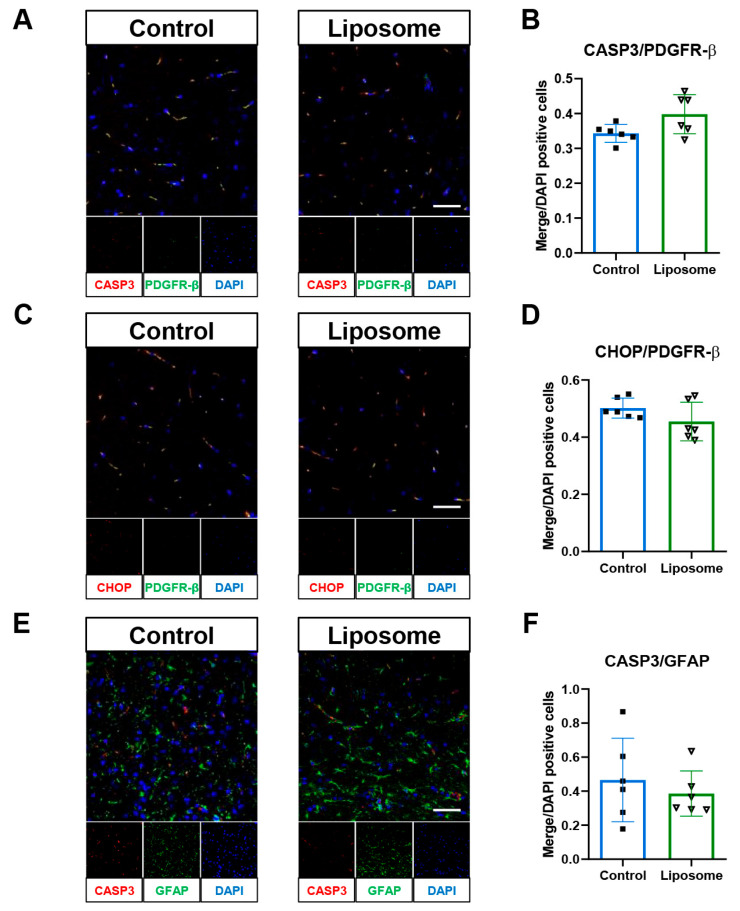
Evaluation of the impact of empty liposomes on cellular stress responses following TBI. (**A**) Immunofluorescence staining of cleaved caspase-3 (CASP3) and PDGFR-β in pericytes. (**B**) Quantification of CASP3/PDGFR-β double-positive cells. (**C**) Immunofluorescence staining of CHOP and PDGFR-β in pericytes. (**D**) Quantification of CHOP/PDGFR-β double-positive cells. (**E**) Immunofluorescence staining of CASP3 and GFAP in astrocytes. (**F**) Quantification of CASP3/GFAP double-positive cells. Immunofluorescence images were acquired from the frontal cortex using confocal microscopy at 200× magnification (scale bar = 50 μm). Across all modalities, empty liposome treatment did not induce measurable changes in BBB leakage, inflammatory gene expression, tight junction protein levels, or pericyte and astrocyte apoptosis after TBI.

Immunofluorescence staining was performed to investigate potential effects on pericyte and astrocyte stress or apoptosis. Cleaved caspase-3/PDGFR-β, CHOP/PDGFR-β, and cCasp3/GFAP signals were all comparable between groups, and quantification showed no significant changes. These results collectively indicate that empty liposomes do not influence pericyte or astrocyte apoptosis or ER stress in the injured brain (Figure 4). These results demonstrate that PEGylated liposomes alone did not alter vascular integrity, inflammatory status, or cellular stress responses in the TBI model.

### 2.4. The LipoStatin Group Showed the Lowest NSS

**Figure 5:**
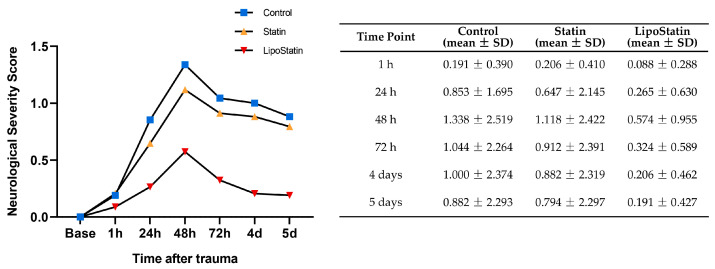
Neurological Severity Score (NSS) assessments in the behavioral cohort (*n* = 34 mice). NSS was measured at 1 h, 24 h, 48 h, 72 h, 4 days, and 5 days post-injury. Animals meeting exclusion criteria (*n* = 6) were excluded from subsequent biochemical assays but retained for behavioral evaluation to represent the complete acute-phase response. Data are presented as mean ± standard deviation.

The clinical condition of injured mice was evaluated by measuring the neurological severity score (NSS) at multiple time points (1 h, 24 h, 48 h, 72 h, 4 days, and 5 days) after trauma (Figure 5). NSS values increased slightly in all groups at 1 h post-injury and peaked at 48 h, coinciding with the termination of repeated TBI. Thereafter, scores declined gradually across all groups. The Control group exhibited the most severe neurological deficits at 48 h (Control, 1.3 ± 2.5; Statin, 1.1 ± 2.4; and LipoStatin, 0.5 ± 0.9), whereas the LipoStatin group consistently showed the lowest NSSs throughout the recovery period. By day 4, NSS scores in the LipoStatin group had returned to near-baseline levels, while the Control and Statin groups continued to exhibit residual neurological impairments (Control, 1.0 ± 2.3; Statin, 0.9 ± 2.3; and LipoStatin, 0.2 ± 0.4). Two-way repeated-measures ANOVA revealed a significant main effect of treatment (F_1,99_ = 4.361, *p* = 0.041) and time (F_1,99_ = 2.150, *p* < 0.001), but no significant treatment × time interaction (F_1,99_ = 0.924, *p* = 0.485). These results indicate that LipoStatin treatment produced a statistically significant overall improvement in neurological function compared with the Control group. However, the temporal pattern of recovery over time was similar across all TBI groups.

### 2.5. LipoStatin Treatment Significantly Decreased BBB Disruption

**Figure 6:**
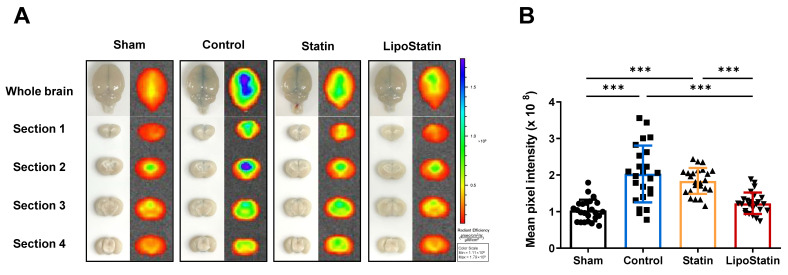
Evaluation of BBB permeability by EB staining and fluorescence imaging on day 5 post-injury in the TBI mouse model. (**A**) After 1 h of EB circulation, perfusion and fixation were performed to capture whole-brain images. Fluorescence imaging using the IVIS^®^ system was used to evaluate EB leakage in whole brains and brain sections, and a color scale indicated leakage areas: blue indicating greater leakage and red indicating reduced leakage. The graph in (**B**) shows the mean pixel intensity from four brain sections per mouse; higher values indicate more severe BBB disruption. The LipoStatin group exhibited significantly reduced EB leakage compared with the Control group, indicating better preservation of BBB integrity (Control vs. LipoStatin: *p* < 0.001, Statin vs. LipoStatin: *p* < 0.001, Welch’s corrected ANOVA with Dunnett’s post hoc analyses). A total of *n* = 24 sections (4 sections per mouse, N = 6 mice) was analyzed. Data were presented as mean ± standard deviation, and statistical significance was denoted as *** *p* < 0.001.

To evaluate recovery of BBB integrity after TBI, we analyzed IVIS^®^ fluorescence images of brain sections (4 sections per mouse; N = 6; Figure 6). The Control group showed the highest pixel intensity in all brain sections, and a particularly noticeable difference was observed in the second section (Figure 6A). The combined mean pixel intensities of the four sections indicated a significant reduction in the LipoStatin group compared with that in the Control and Statin groups (*p* < 0.001, Welch ANOVA; Control vs. LipoStatin: *p* < 0.001, Statin vs. LipoStatin: *p* < 0.001, Dunnett post hoc test; Figure 6B). The LipoStatin group showed recovery close to normal levels on day 5 post-TBI.

### 2.6. LipoStatin Restored Tight Junction Protein Levels

**Figure 7:**
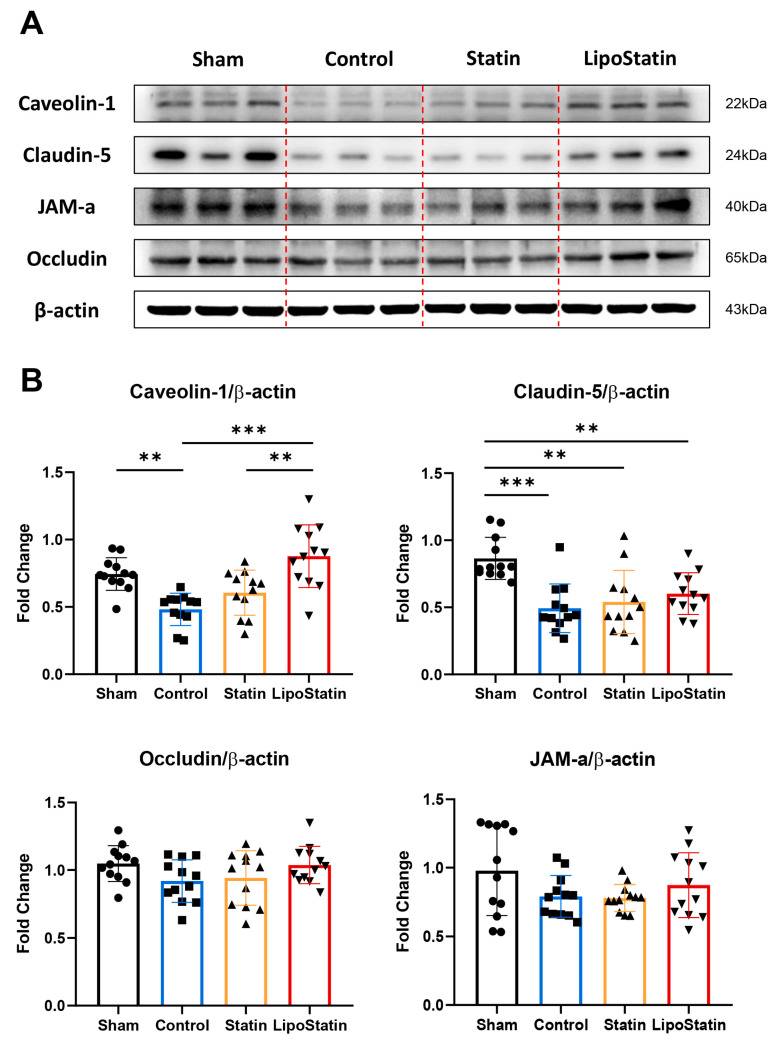
Analyses of TBI-induced changes in protein levels after LipoStatin and atorvastatin treatment. Western blot analyses were performed to measure the protein levels of caveolin-1, claudin-5, JAM-A, and occludin on day 5 post-TBI. β-actin was used for normalization. Group comparisons were performed after quantification of band intensities. (**A**) The protein levels and (**B**) the relative protein levels quantified from the Western blot results. A total of *n* = 12 samples (from N = 6 mice, each analyzed in duplicate) were examined. LipoStatin treatment significantly increased the protein level of caveolin-1 compared with those of the Control and Statin groups, indicating that the BBB integrity was enhanced (Control vs. LipoStatin: *p* < 0.001, Statin vs. LipoStatin: *p* = 0.001, One-way ANOVA with Tukey’s post hoc test). Data were presented as mean ± standard deviation, and statistical significance was indicated as ** *p* < 0.01, and *** *p* < 0.001.

The treatment effect on tight junction protein levels was evaluated by Western blot analyses of caveolin-1, claudin-5, JAM-A, and occludin (Figure 7). For quantification, band intensities were normalized to β-actin to correct for sample loading variability. The protein level of caveolin-1 was significantly higher in the LipoStatin-treated group than in the Control and Statin groups, suggesting the preservation and enhancement of BBB function (*p* < 0.001, one-way ANOVA; Control vs. LipoStatin: *p* < 0.001, Statin vs. LipoStatin: *p* = 0.001, Tukey’s post hoc test). The protein level of claudin-5 was significantly reduced in the TBI mice compared with the sham group (*p* < 0.001, One-way ANOVA; Sham vs. Control: *p* < 0.001, Sham vs. Statin: *p* = 0.001, Sham vs. LipoStatin: *p* = 0.006, Tukey’s post hoc test). JAM-A and occludin protein levels were also reduced in the Control group compared with those in the Sham group; they increased after LipoStatin treatment, but these changes were not statistically significant (Figure 7).

### 2.7. LipoStatin Reduced Inflammatory Cytokine Expression

**Figure 8:**
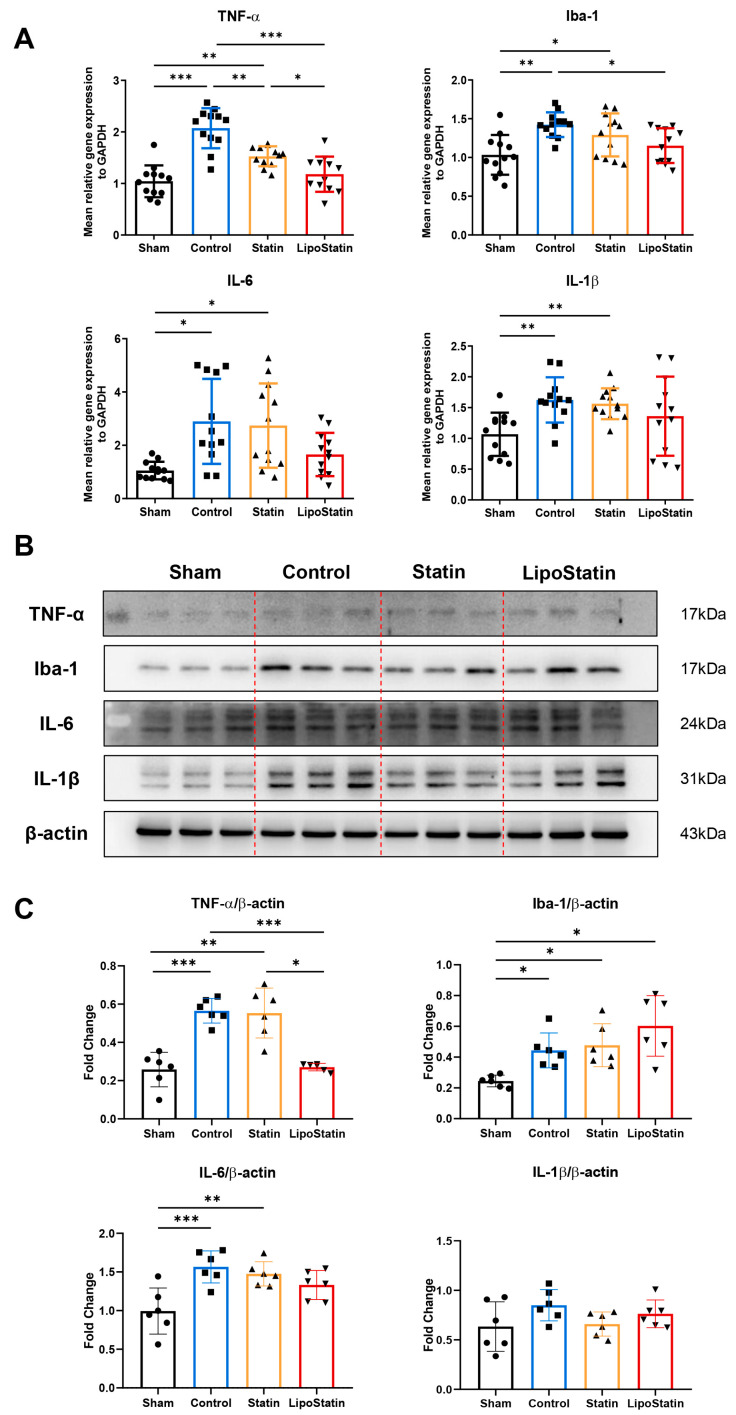
Inflammatory responses in the brain following TBI and their modulation by statin and LipoStatin treatments. (**A**) Quantitative real-time PCR (qRT-PCR) analyses of TNF-α, IL-6, IL-1β, and Iba-1 mRNA levels in brain tissues on day 5 post-TBI. Gene expression was normalized to GAPDH, and data are presented as fold changes relative to the sham group. Each sample was analyzed in duplicate (*n* = 12 from N = 6 mice). (**B**) Western blot analyses of protein levels for the same markers. β-actin was used as the internal loading control. Notably, IL-1β was detected at 31 kDa, corresponding to its precursor form, as the mature 17 kDa form could not be reliably detected under these conditions. (**C**) Densitometric quantification of Western blot bands, normalized to β-actin. TNF-α protein levels were significantly reduced in the LipoStatin group compared to the Control (*** *p* < 0.001, Welch’s ANOVA; Sham vs. Control: *** *p* < 0.001, Sham vs. Statin: ** *p* = 0.006; Control vs. Lipostatin: *** *p* < 0.001, Statin vs. LipoStatin: * *p* = 0.011, Games-Howell test). IL-6 (*** *p* = 0.001, One-way ANOVA; Sham vs. Control: *** *p* = 0.001, Sham vs. Statin: ** *p* = 0.006, Tukey test) and IL-1β showed decreasing trends, though IL-1β was detected only at the 31 kDa precursor form. Iba-1 levels did not differ significantly between groups. Statistical analyses were performed using one-way ANOVA or Welch’s ANOVA with appropriate post hoc tests (Tukey’s or Games–Howell). Results are shown as mean ± SD. * *p* < 0.05, ** *p* < 0.01, *** *p* < 0.001.

To assess neuroinflammatory responses after TBI and the therapeutic effects of treatment, we first evaluated mRNA expression levels of TNF-α, Iba-1, IL-6, and IL-1β using qRT-PCR (Figure 8A). Compared to the sham group, the Control group exhibited significantly elevated expression of all four inflammatory markers, consistent with robust TBI-induced neuroinflammation. LipoStatin treatment significantly downregulated the expression of TNF-α (*p* < 0.001, One-way ANOVA; Control vs. LipoStatin: *p* < 0.001, Tukey’s test) and Iba-1 (*p* = 0.001; Control vs. LipoStatin: *p* = 0.034). The statin group also reduced TNF-α levels relative to control (*p* = 0.001), although the effect was more pronounced in the LipoStatin group (Statin vs. LipoStatin: *p* = 0.048).

To further validate these findings at the protein level, Western blot analyses were performed (Figure 8B,C). Because the available brain tissue per mouse was limited and insufficient to permit repeated extractions and individual-sample analyses, the Western blot was performed once using pooled protein samples per group. TNF-α was detected at its mature 17 kDa form and showed a significant reduction in the LipoStatin group (Welch’s ANOVA *p* < 0.001; Control vs. LipoStatin: *p* < 0.001; Statin vs. LipoStatin: *p* = 0.011). IL-6 and IL-1β proteins showed decreasing trends, consistent with the mRNA results. However, IL-1β was detected only at the 31 kDa precursor form, as the 17 kDa mature band was not clearly visualized under the tested conditions. The absence of detectable mature IL-1β suggests possible post-translational regulation or low abundance below the detection threshold. Iba-1 protein levels showed a modest elevation in the LipoStatin group, contrary to the mRNA results, but no statistically significant difference was observed among groups.

These findings indicate that LipoStatin mitigates TBI-induced inflammatory responses, as evidenced by consistent reductions in TNF-α mRNA and protein levels and trend-level decreases in other markers. The discrepancy observed in Iba-1 highlights the importance of validating both transcriptional and translational changes using complementary methods.

### 2.8. LipoStatin Affected Pericyte and Astrocyte Apoptosis

**Figure 9:**
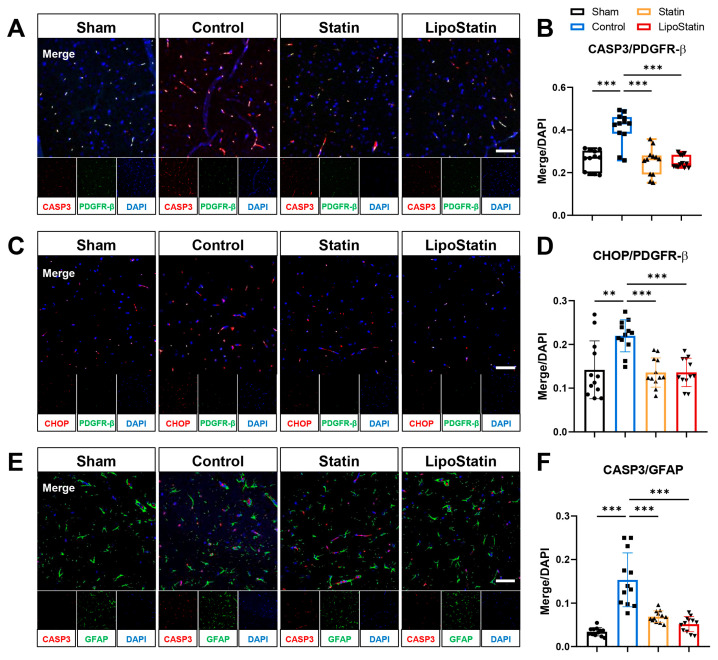
Immunofluorescence analyses of the frontal cortex in the TBI model. (**A**) Immunofluorescence staining of caspase-3 (CASP3, red) and PDGFR-β (green). (**B**) Co-localization of CASP3 and PDGFR-β. (**C**) Immunostaining of CHOP (red) and PDGFR-β (green). (**D**) Co-localization of CHOP and PDGFR-β. (**E**) Immunostaining of CASP3 (red) and GFAP (green). (**F**) Co-localization of CASP3 and GFAP. Immunofluorescence images were captured using confocal microscopy at 200× magnification (scale bar = 50 μm). The right and left cortices were examined, and the results were combined for quantification. Therefore, a total of *n* = 12 (derived from N = 6 mice) was analyzed for each group. Statin and LipoStatin treatments significantly reduced pericyte and astrocyte apoptosis, but LipoStatin showed a stronger protective effect than statin did (one-way ANOVA: CHOP/PDGFR-β, *p* < 0.001; CASP3/GFAP, *p* < 0.001). Post hoc analyses with Tukey’s test showed significant differences in CHOP/PDGFR-β and CASP3/GFAP for Control vs. Statin (*p* < 0.001) and Control vs. LipoStatin (*p* < 0.001). For CASP3/PDGFR-β, which did not follow a normal distribution, a nonparametric Mann–Whitney test was conducted, showing significant differences between Control vs. Statin (*p* < 0.001) and Control vs. LipoStatin (*p* < 0.001). Nonparametric data were presented as box-and-whisker plots showing the interquartile range, minimum, and maximum values. Parametric data were presented as mean ± standard deviation. ** *p* < 0.01, and *** *p* < 0.001.

The co-localization of CASP3 with PDGFR-β, indicative of pericyte apoptosis, was significantly increased in the Control group compared with that in the Sham group (Figure 9A,B). This co-localization was markedly decreased in the statin and LipoStatin treatment groups compared with that in the Control group (*p* < 0.001, Kruskal–Wallis test; Sham vs. Control: *p* < 0.001, Control vs. Statin: *p* < 0.001, Control vs. LipoStatin: *p* < 0.001, Dunn’s post hoc analyses). The co-localization of PDGFR-β and CHOP, which are markers of ER stress in pericytes, significantly increased in the Control group compared with that in the sham group (Figure 9C,D). The co-localization of CHOP/PDGFR-β in the statin and LipoStatin treatment groups was markedly reduced compared with that in the Control group (*p* < 0.001, One-way ANOVA; Sham vs. Control: *p* = 0.001, Control vs. Statin: *p* < 0.001, Control vs. LipoStatin: *p* < 0.001, Tukey’s post hoc test).

The co-localization of CASP3 with GFAP, indicative of astrocyte apoptosis, was significantly increased in the Control group compared with that in the sham group (Figure 9E,F). This co-localization was reduced considerably in the statin and LipoStatin treatment groups compared with that in the control group (*p* < 0.001, One-way ANOVA; Sham vs. Control: *p* < 0.001, Control vs. Statin: *p* < 0.001, Control vs. LipoStatin: *p* < 0.001, Tukey’s post hoc test). Astrocyte apoptosis was reduced by 55.14% with statin and 66.41% with LipoStatin compared with that in the Control group.

## 3. Discussion

**Figure 10:**
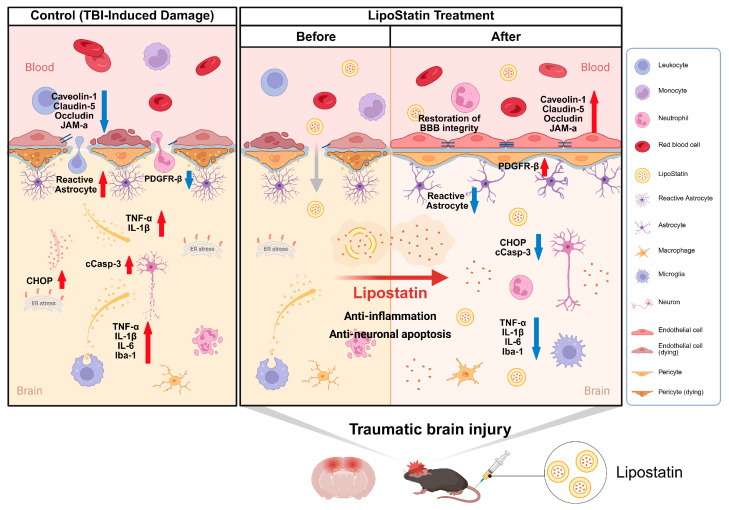
Schematic representation of the putative mechanism of LipoStatin action in TBI. This model is hypothetical and based on indirect findings; direct BBB penetration and tissue accumulation were not assessed in this study.

Our study demonstrated that PEGylated LipoStatin, which likely achieves greater local drug exposure in regions of injury-induced BBB disruption, exerted significant neuroprotective effects in the acute phase of TBI. LipoStatin improved functional outcomes, restored BBB integrity, reduced neuroinflammation, and attenuated pericyte and astrocyte apoptosis (Figure 10). These findings suggest that LipoStatin may be a promising nanocarrier-based therapeutic strategy that enhances drug availability at the injury site during the early post-injury period. Although the overall trajectory of neurological recovery was similar across TBI groups, mice treated with LipoStatin exhibited consistently greater functional improvement, indicating a moderate but distinct behavioral benefit in the acute phase. Our study employed a repetitive TBI model that more closely reflects the cumulative injury patterns observed in clinical populations, such as athletes and military personnel. The efficacy observed under these aggravated pathological conditions suggests potential benefit in mitigating the development of neurological dysfunction. Given the lack of effective pharmacological therapies for TBI, these results support LipoStatin’s translational relevance as a promising therapeutic candidate and highlight the need for further evaluation [2].

The present findings align with previous reports demonstrating that statins can stabilize the BBB and attenuate neuroinflammation following brain injury [7,16]. Consistent with earlier work using LipoStatin, our results further confirm that liposomal delivery enhances drug availability within areas of injury-induced BBB disruption, leading to greater suppression of inflammatory responses and improved therapeutic efficacy compared with free atorvastatin [16]. In particular, LipoStatin markedly reduced BBB disruption and restored caveolin-1 expression more effectively than conventional statin treatment. Although the role of caveolin-1 may vary depending on the injury context, prior studies have shown that its upregulation during the acute phase supports BBB stabilization and vascular repair [30,31,32], findings consistent with our observations. Furthermore, TNF-α levels were substantially lower in the LipoStatin group than in the statin group, further reinforcing the liposomal formulation’s enhanced anti-inflammatory capacity. Collectively, these findings demonstrate that LipoStatin provides stronger BBB-preserving and neuroinflammation-reducing effects than free atorvastatin during the acute phase after repetitive TBI.

The qRT-PCR analyses revealed significant downregulation of pro-inflammatory markers, including TNF-α, Iba-1, IL-6, and IL-1β, in the LipoStatin group. TNF-α protein levels were noticeably decreased, in agreement with mRNA findings, whereas IL-6 and IL-1β exhibited only modest declines in the corresponding Western blot. For IL-1β, only the 31 kDa precursor band was detected, and the mature 17 kDa band was absent, suggesting insufficient cleavage, low abundance, or limitations in methodological sensitivity. Similarly, Iba-1 protein levels did not fully parallel their transcriptional trends. Such discrepancies have been reported in previous studies. They may arise from regional heterogeneity, post-transcriptional regulation, or technical constraints— particularly the single-run Western blot performed on whole-hemisphere homogenates, which may dilute localized inflammatory signals [33,34,35]. Additional protein-level validation using ELISA or region-specific, replicated Western blot assays would further strengthen these findings [36,37]. Despite these limitations, the consistent suppression of mRNA levels, together with improvements in NSSs and IVIS^®^ outcomes, supports the anti-inflammatory potential of LipoStatin.

At 48 h post-injury, all experimental groups exhibited aggravated neurological deficits, as reflected by elevated NSS values. This neurological deterioration corresponds to the well-established peak of acute secondary injury processes following TBI—including BBB breakdown, vasogenic edema, oxidative stress, and robust neuroinflammatory activation—which typically culminate within the first 48 h before reparative mechanisms emerge [38,39,40]. From a pharmacological standpoint, PEGylated LipoStatin exhibits a sustained-release profile and delayed central nervous system penetration [41,42]; therefore, its therapeutic effects may not fully manifest at this early time point. The worsening of NSS values could thus be interpreted as a physiological consequence of secondary injury progression rather than an indication of treatment inefficacy [38,41,43]. This explanation aligns with prior findings showing that statin-mediated anti-inflammatory and barrier-restorative mechanisms become more prominent after the immediate acute phase.

Therefore, we investigated the effects of LipoStatin on apoptosis in pericytes and astrocytes after the acute phase of TBI at the cellular level, and confirmed that LipoStatin promoted the survival of key BBB-associated cell types [44]. Specifically, immunofluorescence analyses revealed reduced apoptosis and ER stress, as evidenced by decreased colocalization of caspase-3 and CHOP with PDGFR-β and GFAP, established markers of cellular injury in TBI models [45,46]. Given the pronounced spatial differences between injured and uninjured areas in focal TBI, immunofluorescence provides crucial regional information on cell-specific vulnerability and colocalization patterns [47,48]. The immunofluorescence findings were consistent with NSS, IVIS^®^, and qRT-PCR results, strengthening the validity of the observed neuroprotective effects. Although protein-level confirmation of caspase-3 and CHOP could not be obtained by Western blot in the current study, convergent evidence from immunofluorescence and behavioral/molecular outcomes supports a coherent biological interpretation of LipoStatin’s protective actions [49,50]. These results suggest that LipoStatin modulates survival pathways in multiple components of the neurovascular unit, thereby contributing to broader neurovascular stability and functional recovery [44]. Maintaining the viability of pericytes and astrocytes is essential for preserving BBB integrity and promoting neurological recovery after TBI [51,52,53,54,55]. Taken together, our findings also highlight the potential applicability of LipoStatin for neurological disorders involving apoptosis and ER stress beyond TBI [56,57].

The beneficial outcomes of LipoStatin observed in this study are likely attributable to the improved pharmacokinetic properties and enhanced local drug exposure achieved with the PEGylated liposomal formulation. Unlike conventional statins, which exhibit low oral bioavailability due to rapid gastrointestinal clearance, limited solubility, and poor permeability [58], intravenously administered formulations may provide more reliable systemic levels. In line with previous preclinical findings, the total daily dose of 10 mg/kg used in this study appears sufficient to exert neuroprotective effects when delivered intravenously [59]. Both free atorvastatin and LipoStatin reduced astrocyte apoptosis; however, the improvement tended to be greater in the LipoStatin group, although this difference did not reach statistical significance. This trend may reflect the structural advantages of the liposomal carrier, which enables prolonged systemic circulation and enhanced distribution to regions of injury-induced BBB permeability rather than active molecular targeting [16,60]. Our findings also suggest the potential utility of LipoStatin in other neurological diseases characterized by BBB disruption, neurovascular instability, and inflammation. Prior studies in ischemic stroke and intracerebral hemorrhage models demonstrated similar benefits of liposomal atorvastatin formulations, including improved neurovascular protection and functional outcomes [16,61]. By enhancing drug availability in areas of BBB compromise, LipoStatin may offer therapeutic advantages across a broad range of neurovascular disorders and could outperform conventional statin therapies in settings where localized vascular injury plays a central role [62,63].

To confirm the biodistribution of LipoStatin, we aimed to directly assess the brain concentration of atorvastatin using liquid chromatography–tandem mass spectrometry (LC–MS/MS) analyses of brain tissue following intravenous administration. Atorvastatin was not detected in the Sham, Control, or empty Liposome groups, confirming the absence of nonspecific background signal. In contrast, low nanogram concentrations were measurable in both the Statin and LipoStatin groups, indicating that systemic dosing might enable the drug to cross the BBB and enter the TBI lesion, albeit at low levels. Analyses of residual proportions demonstrated that LipoStatin yielded a markedly higher percentage of residual drug in the brain 24 h after injection, just before the next infusion, compared with free statin. Specifically, the estimated residual fraction of atorvastatin 48 h after injection was approximately 4-fold higher in the LipoStatin group than in the Statin group, consistent with the enhanced drug availability and prolonged distribution characteristics of PEGylated liposomal formulations. These pharmacokinetic observations suggest that LipoStatin may support extended therapeutic exposure in regions of traumatic injury and align with the functional and histological improvements observed in LipoStatin-treated mice, reinforcing the formulation’s therapeutic potential.

To examine whether the liposomal carrier itself could influence biological outcomes, we performed additional control experiments comparing empty PEGylated liposomes with saline-treated animals. Consistent with previous studies reporting that PEGylated liposomes alone do not substantially alter infarct volume, apoptosis, or inflammatory responses in cerebral ischemia–reperfusion injury models, empty liposomes in our study did not influence measurable effects on BBB integrity, inflammatory markers, or tight-junction protein expression [28,64]. These findings support the conclusion that the neuroprotective effects observed with LipoStatin are attributable to atorvastatin delivery rather than nonspecific actions of the liposomal carrier.

Despite the promising findings, several limitations should be acknowledged. First, this study utilized a rodent repetitive TBI model, which inherently limits direct clinical translation. Nevertheless, the weight-drop paradigm remains a widely accepted and reproducible model for evaluating early neurovascular responses to injury. PEGylated liposomes, including LipoStatin, have shown translational potential in other neurological disease models; however, additional studies are needed to evaluate large-scale manufacturing feasibility, cost-effectiveness, and regulatory considerations in the context of TBI [65,66]. Second, our investigation primarily focused on short-term outcomes assessed on day 5, with additional cellular analyses performed on day 12. While this timeframe effectively captured acute cerebrovascular and inflammatory responses, it did not permit evaluation of long-term functional recovery or late-phase neuropathology. As such, the present results reflect acute-phase effects, and the durability of these benefits remains uncertain. Future studies incorporating extended observation periods are required to determine whether early improvements translate into persistent functional recovery and mitigation of chronic sequelae, such as cognitive impairment or neurodegeneration. Third, although we were able to directly quantify atorvastatin levels in brain tissue using LC–MS, providing partial evidence of cerebral drug exposure, these analyses may not clarify the spatial distribution of the drug or the liposomal carrier’s penetration. LC–MS reflects whole-hemisphere concentrations and cannot determine whether drug accumulation was localized to the injured region or uniformly distributed. Moreover, the biodistribution of the liposomal nanocarrier has not been verified [61,67]. Future studies using radiolabeled liposomes, autoradiography, or advanced imaging modalities will be beneficial to characterize the regional distribution, cellular uptake, and pharmacokinetics of LipoStatin more comprehensively. Fourth, the manually operated device used to induce TBI may introduce variability in impact force and angle. To minimize this limitation, we adhered to strict experimental procedures, implemented long-term operator training, and randomized treatment assignments after injury induction. Nevertheless, automated impact systems may further enhance reproducibility in future studies. Lastly, we did not investigate whether the observed neuroprotective effects of LipoStatin are mediated through specific downstream signaling pathways (e.g., PI3K/AKT, NF-κB) [68]. Cross-modal validation was also limited: qRT-PCR, IF, and Western blot were performed on different tissue fractions and time points. The Western blot for inflammatory markers was performed only once using pooled ipsilateral tissue, potentially diluting lesion-specific signals. Notably, mature IL-1β protein was undetectable despite significant mRNA suppression, underscoring the technical limitations of a single-run Western blot for low-abundance cytokines. Additionally, IF quantitative protein assays did not corroborate results, they may reflect changes in localization rather than expression levels [69]. Future studies incorporating synchronized, replicated protein assays (e.g., ELISA or region-specific Western blot) and mechanistic pathway analyses are needed to confirm the basis of LipoStatin’s neuroprotective effects.

In conclusion, this study provides the first evidence that LipoStatin can enhance neuroprotection after TBI by restoring functional impairment, stabilizing the BBB, reducing neuroinflammation, and preventing apoptosis of pericytes and astrocytes within the BBB (Figure 10). These findings suggest that LipoStatin could be a promising therapeutic candidate for TBI. Further studies are needed to address scalability, cost-effectiveness, and regulatory challenges before clinical application.

## 4. Materials and Methods

### 4.1. Experimental Animals

Adult male C57BL/6 mice (age = 8 weeks; weight = 22–26 g) were purchased from Samtako Bio Korea Co., Ltd. (Seoul, Republic of Korea). They were housed under a 12-h light–dark cycle with free access to food and water. All animal protocols were conducted in accordance with the guidelines of Chonnam National University for the care and use of laboratory animals and were approved by the Institutional Animal Care and Use Committee (IACUC) (Approval No. CNUHIACUC-19131, Approval date: 13 December 2019). All experimental procedures were also performed in compliance with the United States National Institutes of Health (NIH) guidelines for the humane care and use of laboratory animals. To minimize animal suffering, appropriate anesthetics (e.g., isoflurane) were administered during the procedure to ensure animal well-being.

### 4.2. Traumatic Brain Injury Model

**Figure 11:**
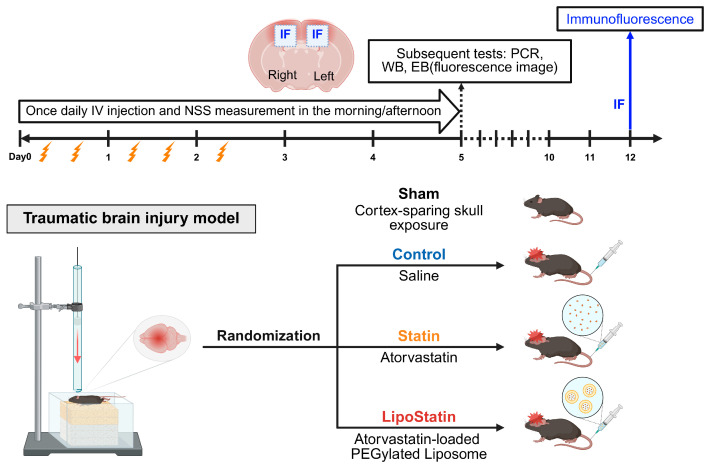
Overview of the study protocol using a traumatic brain injury model to assess the efficacy of various treatments.

Each mouse was anesthetized with 3% isoflurane in a 30/70 mixture of oxygen and nitrous oxide for induction and maintained with 2% isoflurane while positioned in a stereotaxic frame. During surgery and recovery, the animals’ body temperature was maintained at 36.5 ± 0.2 °C with a temperature-controlled heating pad. TBI was induced using a weight-drop device based on the Marmarou method [70,71]. The scalp was vertically incised to expose the skull, and the mouse’s head was secured to the base of the impact device. Five consecutive head impacts were delivered at 12-h intervals using a 120 g weight dropped from a height of 60 cm onto a cone positioned over the exposed skull, with an impact-absorbing sponge placed beneath the impact area to minimize fatal outcomes. Immediately after the impact, the scalp was closed with Durapore surgical tape (3M) or manually with forceps. The incision sites generally healed rapidly without complications. This protocol was selected to model repetitive TBI relevant to sports-related concussions and military operations [72,73,74]. After surgery, the mice were returned to their cages and allowed free access to food and water. A total of 96 mice were randomly assigned to four treatment groups (*n* = 6 per group): sham surgery, control, statin, and LipoStatin groups. In each group, the mice were intravenously injected with saline, atorvastatin, or LipoStatin at a total daily dose of 10 mg/kg for up to 5 days post-TBI. Given the minimal injection volume tolerated by mice and the potential for residual or leaking fluid during intravenous administration, the total daily dose of 10 mg/kg was selected based on prior preclinical studies [75,76]. The mice in the sham surgery group underwent the same scalp incision procedure but did not receive cortical impact (Figure 11). To evaluate neurological outcomes, a separate behavioral cohort (*n* = 34 mice) was established to assess the Neurological Severity Score (NSS) at multiple time points (1 h, 24 h, 48 h, 72 h, 4 days, and 5 days post-injury). This larger sample size accounted for potential attrition and allowed balanced assignment for downstream analyses. Animals exhibiting severe acute injury (NSS > 9 immediately post-TBI), death within 5 days, or >25% body-weight loss were excluded before terminal assays. Based on these predefined exclusion criteria, six mice were excluded (two each from the Control, Statin, and LipoStatin groups), leaving *n* = 6 mice per group (total *n* = 24) for biochemical and histological analyses (Western blot, qRT-PCR, immunofluorescence, and IVIS^®^). Random allocation to treatment groups was performed after injury to minimize baseline bias. Some biochemical assays were performed in duplicate, yielding a sample size of *n* = 12 per group.

### 4.3. Preparation and Characterization of LipoStatin

As previously reported, liposomes were synthesized via the film hydration [16]. Briefly, a lipid mixture of 1,2-distearoyl-sn-glycero-3-phosphoethanolamine (DSPE-PEG2000, MW ≈ 2000 Da), dipalmitoylphosphatidylcholine (DPPC), atorvastatin calcium (dissolved in methanol), and cholesterol (dissolved in chloroform) was combined, vortexed for 2 min, and evaporated under vacuum at 25 °C to form a thin lipid film. The film was hydrated with 1 mL of PBS and stirred at 60 °C for 5 min to form liposomes. The dispersion was sonicated using a probe sonicator for 5 min, and unencapsulated atorvastatin was removed by dialysis (6–8 kDa MWCO; Spectra/Por, Spectrum Labs Inc., Phoenix, AZ, USA) against distilled water at 4 °C for 12 h.

Physicochemical characterization was carried out as follows. The hydrodynamic diameter, PDI, and zeta potential were measured using a Zetasizer Nano ZS (Malvern Instruments Ltd., Malvern, UK; software version 7.01) based on Dynamic Light Scattering (DLS). Measurements were conducted in phosphate-buffered saline (PBS) at 25 ± 0.5 °C using a 90° detection angle, with triplicate (*n* = 3) measurements per sample.

Encapsulation efficiency was determined via ultrafiltration (Amicon Ultra-4, 10 kDa MWCO) followed by HPLC quantification at 244 nm. Stability and batch reproducibility were confirmed by comparing particle size and PDI immediately after preparation and after 24 h of storage at 4 °C across three independent batches. Transmission Electron Microscopy (TEM) was performed using a JEM-2010 microscope (JEOL, Tokyo, Japan) operated at 200 kV. Liposomes were negatively stained with Synomag (0.5 mg/mL) and air-dried at room temperature on a 200-mesh carbon-coated copper grid before imaging.

### 4.4. LC-MS/MS Analyses of Atorvastatin in Brain Tissue

Mouse brains were collected following euthanasia, and the whole brain (excluding the cerebellum) was carefully dissected and rinsed with ice-cold phosphate-buffered saline (PBS) to remove residual blood. After gently blotting on sterile gauze, each brain was weighed and stored at –80 °C.

For LC–MS/MS sample preparation, frozen brains were thawed on ice and homogenized in 1 mL of 100% methanol using a mechanical homogenizer. The homogenates were centrifuged at 13,000 rpm for 10 min at 4 °C, and the supernatant (approximately 800 µL) was collected for LC–MS/MS analyses.

Atorvastatin concentrations were quantified using LC–MS on a Shimadzu LC-40A system (Shimadzu Co., Kyoto, Japan) equipped with a dual solvent pump (LC-40Dx3), an autosampler (SIL-40Cx3), and a column oven (CTO-40C). The LC system was coupled to an AB SCIEX QTRAP^®^ 4500 mass spectrometer (AB Sciex Pte. Ltd., Redwood City, CA, USA) equipped with a Turbo Ion Spray electrospray ionization (ESI) source operating in positive ion mode.

Mass spectrometric detection was performed in multiple reaction monitoring (MRM) mode with transitions of *m*/*z* 559.3 → 440.1 for atorvastatin and *m*/*z* 482.3 → 258.2 for rosuvastatin (internal standard, IS). The ion spray voltage was 5000 V, with curtain gas (CUR) at 35 psi, ion source gas 1 (GS1) at 50 psi, ion source gas 2 (GS2) at 50 psi, and source temperature at 450 °C.

Chromatographic separation was achieved on a Gemini C18 column (5 µm, 110 Å, 50 × 2.0 mm; Phenomenex) equipped with a Gemini C18 guard cartridge (4.0 × 2.0 mm) and maintained at 40 °C. The mobile phase consisted of (A) 0.1% formic acid in water and (B) 0.1% formic acid in acetonitrile, delivered at 0.3 mL/min under a gradient program: 0–0.5 min, 20% B; 0.5–1 min, 70% B; 1–2 min, 70% B; and 2.0–2.1 min, 20% B. The total run time was 4 min, and the autosampler was maintained at 15 °C. Retention times were approximately 2.2 min for atorvastatin and 1.8 min for rosuvastatin.

Calibration standards were prepared in 50% methanol at concentrations of 0.005, 0.02, 0.05, 0.2, 0.5, and 2 ng/mL, and stock solutions (1000 µg/mL in methanol) of both analytes were freshly prepared and stored at 4 °C. For quantitative analyses, 400 µL of a 50% methanol-diluted sample was mixed with 10 µL of an internal standard (rosuvastatin at 100 ng/mL in 50% methanol), vortexed, and 5 µL of the mixture was injected into the LC–MS/MS system. Quantification was based on a calibration curve constructed from the ratio of the atorvastatin peak area to that of the internal standard. Data acquisition and analyses were performed using SCIEX OS 3.1 software (AB Sciex, Framingham, MA, USA).

### 4.5. Neurological Severity Score (NSS)

The initial neurological impairment assessed 1 h post-TBI is a robust indicator of TBI severity [71,77]. NSS was evaluated in a dedicated behavioral cohort of 34 mice, independent of the biochemical assay subset, to capture functional recovery patterns during the acute phase. In the scoring system, 10 clinical parameters related to motor function, alertness, and physiological behavior were evaluated during 5 days after TBI: (i) exit circle, the mouse’s ability and initiative to exit a 30 cm diameter circle within 3 min; (ii) mono- or hemiparesis, paresis of the upper and/or lower limb on the contralateral side; (iii) straight walk, the mouse’s alertness and motor ability to walk straight upon being placed on the floor; (iv) startle reflex, an innate reflex by which the mouse startles and jumps in response to a loud hand clap; (v) seeking behavior, a physiological behavior indicating “interest” in the environment; (vi) beam balancing, the ability to balance on a 7 mm wide beam for at least 10 s; (vii) grip on a round stick, the ability to grip a 5 mm wide beam for at least 10 s; and (viii–x) beam walk—3, 2, and 1 cm, the ability to cross a 30 cm long beam of decreasing widths (3, 2, and 1 cm), representing increasing difficulty levels [71]. A point was assigned for the absence of a tested reflex or inability to perform the tasks, and no points were awarded for task completion. A maximal NSS of 10 points indicated profound neurological dysfunction, reflecting the failure to perform any tasks. Using this method, a scientifically grounded, medically relevant framework could be used to assess the degree of neurological impairment following TBI (Figure 11).

### 4.6. Evans Blue Staining and Fluorescence Imaging

The BBB integrity was evaluated as previously described on day 5 post-TBI [78] (Figure 11). Evans blue dye (EB, 2% *w*/*v* in saline) was injected intravenously and allowed to circulate for at least 60 min. Under isoflurane anesthesia, the chest was opened, and the intravascular dye was removed by perfusing saline through the left ventricle until a colorless perfusate was obtained from the right atrium. The brain was extracted and fixed in 4% paraformaldehyde for 24 h. After fixation, EB staining was visualized through fluorescence analyses. The extracted brain was sectioned coronally into 2 mm-thick slices, and vascular permeability was assessed by measuring EB extravasation using an in vivo imaging system (IVIS^®^ lumina S5, PerkinElmer, Waltham, MA, USA). Ex vivo images were obtained for the whole brain and the 2 mm thick tissue slices using the following parameters: excitation filter, 600 nm; emission filter, 710 nm; exposure time, 0.5 s; binning, 8; f-stop, 2; and field of view, 21 cm. Regions of interest (ROI) in the brain and brain slices were analyzed by observing EB fluorescence. The extent of EB leakage between groups was quantified by measuring the total pixel intensity.

### 4.7. Immunoblot Analyses and Quantitative Reverse Transcription Polymerase Chain Reaction (qRT-PCR)

The mouse brains (*n* = 6 per group) were used to prepare protein extracts. Western blotting was performed in duplicate to evaluate the protein levels of tight junction and regulatory proteins such as caveolin-1 (1:1000, Abcam, ab2910, Cambridge, UK), claudin-5 (1:1000, Abcam, ab15106, UK), JAM-A (1:500, Abcam, ab52647, UK), occludin (1:1000, Thermo Scientific, 71-1500, Waltham, MA, USA), IL-1β (1:1000, Cell Signaling, 12703s, Danvers, MA, USA), IL-6 (1:1000, Cell Signaling, 12912s, USA), Iba-1 (1:1000, Cell Signaling, 17198s, USA) and TNF-α (1:1000, Cell Signaling, 11948s, USA) in the BBB on day 5 post-TBI. In immunoblot analyses, β-actin (1:2000, Santa Cruz Biotechnology, sc-47778, Dallas, TX, USA) was used as an internal control. The intensity of each target protein band was normalized to the corresponding β-actin band intensity to correct for loading variability.

Total RNA was extracted from the brain, and cDNA was synthesized. qRT-PCR was performed in duplicate to measure the expression levels of inflammatory cytokines such as tumor necrosis factor-alpha (TNF-α), ionized calcium-binding adaptor molecule 1 (Iba-1), interleukin-1 beta (IL-1β), and interleukin-6 (IL-6) on day 5 post-TBI (Figure 11). Glyceraldehyde-3-phosphate dehydrogenase (GAPDH) was used as a reference gene for normalization.

### 4.8. Double Immunofluorescent Staining

Slides were immersed in xylene for 30 min and rehydrated in graded alcohols (100% ethanol and 75% ethanol, five changes each, 1 min per change) to remove paraffin. They were rinsed with PBS for 5 min, and antigens were retrieved in a 10 mM citrate buffer with 2 mM EDTA and 0.05% Tween 20 at pH 6.0 in a pressure cooker for 15 min. Afterward, they were cooled for 20 min and rinsed again with PBS for 15 min.

Tissues were permeabilized in a 3% H_2_O_2_–methanol solution for 15 min, then rinsed with PBS for an additional 15 min. Slides were incubated in 3% bovine serum albumin for 15 min, dried, and placed in a humid chamber to reduce nonspecific binding. Our preliminary results showed that cellular apoptosis was most severe 12 days after TBI, so we performed immunofluorescence analyses on day 12 post-TBI (Figure 11). The following primary antibodies were used at 4 °C overnight to evaluate cellular apoptosis: mouse anti-caspase-3 (1:100, Thermo Fisher Scientific, MA3100, Waltham, MA, USA) and mouse anti-CHOP (1:100, Cell Signaling Technology, #2895, Danvers, MA, USA). After the slides were washed with PBS for 15 min, they were incubated with secondary antibodies, including rabbit anti-PDGFR-β (1:100, Thermo Fisher Scientific, MA5-15143) as a pericyte marker and rabbit anti-GFAP (1:200, Thermo Fisher Scientific, PA1-10019) as an astrocyte marker, for 4 h at room temperature. After another PBS rinse, the slides were labeled with Alexa Fluor 594- and Alexa Fluor 488-conjugated IgG for mouse and rabbit antibodies, respectively, at a 1:400 dilution for 1 h at room temperature. All washes and incubations were performed in the dark.

The slides were stained with DAPI (1:1000, Invitrogen, cat#P36935, Waltham, MA, USA) for 10 min, washed with 1X TBST and distilled water for 15 min, air-dried, covered with a cover slip, and sealed with nail polish. They were stored in a dark box at room temperature and observed under a Zeiss LSM-510 microscope with appropriate filters. Images were analyzed using Adobe Photoshop version 6, and double-labeled cells were quantified using an optical classifier method. The number of merged cells was divided by the total number of cells to calculate the percentage of double-labeled cells.

### 4.9. Statistical Analysis

Data were statistically analyzed using IBM SPSS Statistics 29. Parametric data were examined using one-way ANOVA and Tukey’s post hoc test for multiple comparisons. Normality and homogeneity of variances were assessed using the Shapiro–Wilk and Levene’s test, respectively. Results were expressed as means ± standard deviations (SD). Welch’s ANOVA was applied to data with unequal variances, followed by Dunnett’s post hoc test. Nonparametric data were analyzed using the Kruskal–Wallis test and reported as medians [interquartile ranges]. A repeated-measures ANOVA was performed on NSS data, with time and treatment as within-subject factors. All measurements were performed by an observer blinded to the treatment groups. Results with *p* < 0.05 were considered statistically significant: * *p* < 0.05, ** *p* < 0.01, and *** *p* < 0.001.

## Data Availability

The original contributions presented in this study are included in the article. Further inquiries can be directed to the corresponding authors.

## Abbreviations

TBI	traumatic brain injury
PEG	polyethylene glycol
BBB	blood–brain barrier
NSS	neurological severity score
HMG-CoA	3-hydroxy-3-methyl-glutaryl-coenzyme A
DSPE-PEG	1,2-distearoyl-sn-glycero-3-phosphoethanolamine
DPPC	Dipalmitoylphosphatidylcholine
EB	Evans blue dye
IVIS	in vivo imaging system
ROI	Regions of interest
qRT-PCR	Quantitative Reverse Transcription Polymerase Chain Reaction
TNF-α	tumor necrosis factor-alpha
Iba-1	ionized calcium-binding adaptor molecule 1
IL-1β	interleukin-1 beta
IL-6	interleukin-6
GAPDH	Glyceraldehyde-3-phosphate dehydrogenase
SD	standard deviation
rmTBI	repetitive mild traumatic brain injury
